# Exploring the link between metabolic syndrome and risk of dysmobility syndrome in elderly population

**DOI:** 10.1371/journal.pone.0207608

**Published:** 2018-12-11

**Authors:** Yuan-Yuei Chen, Tung-Wei Kao, Chung-Ching Wang, Ying-Jen Chen, Chen-Jung Wu, Wei-Liang Chen

**Affiliations:** 1 Department of Internal Medicine, Tri-Service General Hospital Songshan Branch, Taipei, Taiwan, Republic of China; 2 Division of Family Medicine, Department of Family and Community Medicine, Tri-Service General Hospital, and School of Medicine, National Defense Medical Center, Taipei, Taiwan, Republic of China; 3 Division of Geriatric Medicine, Department of Family and Community Medicine, Tri-Service General Hospital, and School of Medicine, National Defense Medical Center, Taipei, Taiwan, Republic of China; 4 Graduate Institute of Clinical Medical, College of Medicine, National Taiwan University, Taipei, Taiwan, Republic of China; 5 Department of Ophthalmology, Tri-Service General Hospital, National Defense Medical Center, Taipei, Taiwan, Republic of China; 6 Division of Family Medicine, Department of Community Medicine, Taoyuan Armed Forces General Hospital, Taoyuan, Taiwan, Republic of China; International University of Health and Welfare, School of Medicine, JAPAN

## Abstract

Dysmobility syndrome (DMS) was considered as a comprehensive approach to evaluate the condition of musculoskeletal system and adverse health problems in older population. The objective of our study was to examine the association between metabolic syndrome (MetS) and DMS in a U.S. adult population. 1760 eligible participants from the National Health and Nutrition Examination Survey (NHANES) 1999–2002 were enrolled in the study. The criteria of DMS consisted of six domains including increased body fat, declined muscle mass, reduced muscle strength, osteoporosis, slow gait speed, and balance problem. A multivariate regression analysis was investigated to clarify the relationship among MetS and its components and DMS. A positive association between increased number of MetS components and the presence of DMS achieved significance (β = 0.142, 95%CI = 0.035, 0.249, p = 0.009). Among the components of MetS, hyperglycemia had a central place in the DMS after adjustment of clinical variables (β = 0.083, 95%CI = 0.030, 0.136, p = 0.002). Notably, insulin resistance assessed by homeostatic model assessment (HOMA-IR) was correlated to increased body fat (r = 0.092, p<0.05), osteoporosis (r = -0.105, p<0.05) and balance (r = 0.105, p<0.05) among these participants with MetS. Our study demonstrated a strong relationship between DMS and the presence of MetS and its components in elderly population, highlighting a possible mechanism through insulin resistance.

## Introduction

In the recent decades, several medical terms were used to address the association between musculoskeletal system, physical function, and body composition for evaluating the decline in health and aging. However, not only the term “sarcopenia[1]” but also “osteosarcopenic obesity[2]” only focus on the aspects of muscle, bone condition and obesity that was too narrow in both identifying people at risk of decline and to inform intervention development and implementation. Recently, dysmobility syndrome (DMS), reported by Binkley et al[3], was a potential and developing territory to describe as a multicomponent classification which considering the interaction across such health conditions and incorporating wide range of factors to evaluate comprehensively the adverse outcomes in elderly people. Looker et al. had reported that patients with DMS had increased mortality risks among older population [4].

There had been a substantial body of studies in the literature concerning the relationship among MetS, frailty, and sarcopenia. MetS was a cluster factors contributing to multiple chronic diseases and increased risk of all-cause and cardiovascular mortality[5] and was prevalent in U.S. adults. The presence of metabolic syndrome (MetS) played an important role in several adverse health conditions, such as low lean muscle mass, reduced muscle strength, osteoporosis, central obesity, functional dependency, and mobility limitation. To date, the research was still at an early stage in the application of DMS to clinical setting, not to mention a paucity of literature associated with MetS. In the present study, we hypothesized that MetS might contribute to the development of DMS through several underlying mechanisms. Our aim was to investigate the association between MetS and DMS using the population-based dataset.

## Methods

### Participants and study design

The study sample was derived from the National Health and Nutrition Examination Survey (NHANES) 1999–2002, which was a multistage and population-based survey consisted of detailed home interview and a health examination for collecting information on the health and nutrition status to obtain a representative sample of the United States noninstitutionalized civilian. All measurements in NHANES were approved by the National Center for Health Statistics (NCHS) Institutional Review Board (ethic approval code: Protocol #98–12), and written informed consent was obtained from all subjects.

**Fig 1:**
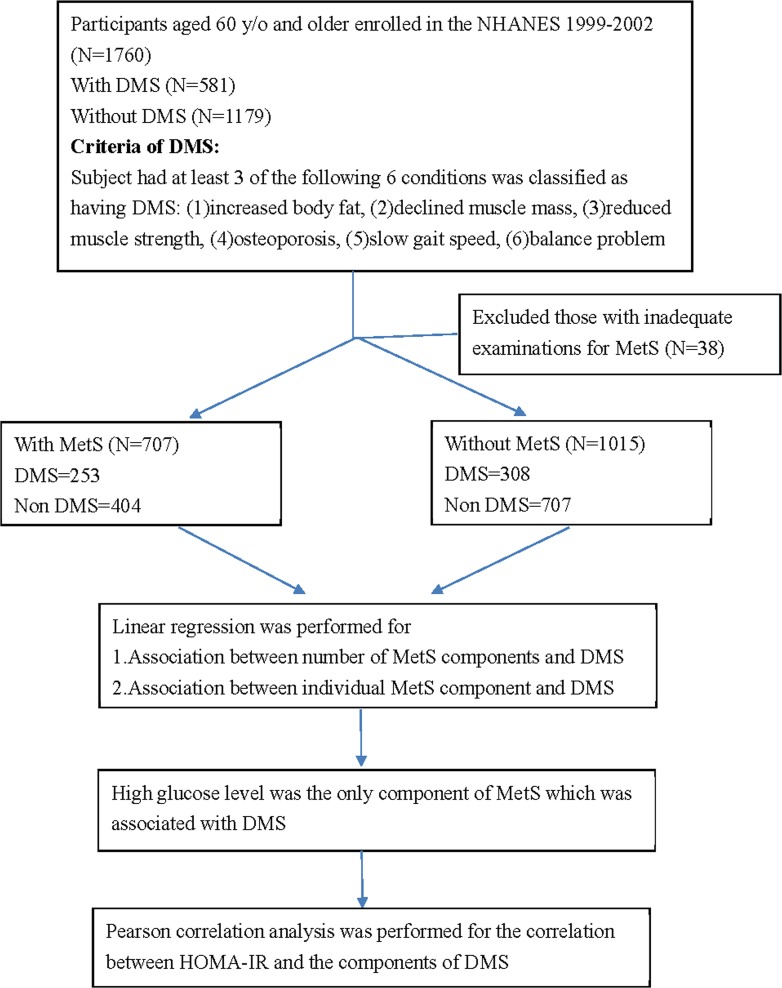
Flow chart of our study.

According to the flow chart of the study (Fig 1), 1760 eligible participants aged 60 years old and older were obtained from the NHANES 1999–2002 who were with (n = 581) and without (n = 1179) DMS. After excluding those with inadequate examinations for MetS, we divided these participants into 2 groups based on the presence of MetS, respectively. There were 707 subjects with MetS (DMS: 253, non-DMS: 454) and 1015 (DMS: 308, non-DMS: 707) without MetS.

### Definition of MetS

According to the criteria defined by National Heart, Lung, and Blood Institute[6], a participant was regarded as with MetS when manifesting three or more of the following five criteria: waist circumference (WC) ≧102 cm in men, ≧89 cm in women; a high triglyceride (TG) level≧150 mg/dL; high density lipoprotein cholesterol (HDL-C)<40 mg/dL in men, <50 mg/dL in women; systolic and diastolic blood pressure (SBP/DBP)≧130/85 mmHg, or use of antihypertensive drugs or a past history of hypertension (HTN); fasting plasma glucose (FPG)>100 mg/dL, or a past history of type 2 diabetes mellitus (DM), or current use of antidiabetic agents.

### Definition of DMS

Binkley et al. presented the original definition of DMS included six domains: increased body fat, declined muscle mass, reduced muscle strength, osteoporosis, slow gait speed, and balance problem, which subject had at least 3 of the following 6 conditions was classified as having DMS[3]. We applied the method and cutoff points in the previous study reported by Binkley et al. to describe the complicated conditions in the elderly, such as increased body fat, declined muscle mass, and low gait speed. The cutoff points of reduced muscle strength, osteoporosis and balance problem were defined by the respective measurements in the NHANES.

### Total body composition

Total body dual-energy X-ray absorptiometry (DXA) scan, conducted by Hologic QDR 4500A fan-beam densitometers (Hologic, Inc., Bedford, Massachusetts), was used to detect the body fat and appendicular lean mass. High body fat was defined by the present published cutoff points which male was over 30 percent and female were over 40 percent[7]. The published values of low lean mass were originally reported by Baumgartner et al. that below 5.45 and 7.26 kg/m2 for females and males, respectively[8].

### Osteoporosis

Based on the WHO classification, the diagnostic criteria for osteoporosis was defined as T-score of less than or equal to −2.5 at the lumbar spine, femoral neck, or total proximal femur by the bone mineral density (BMD) test[9]. BMD at the lumbar spine subregion was conducted in our study because the osteoporosis prevalence and the occurrence of fracture was comparable to BMD estimating from a devoted AP view of lumbar spine scan[10]. Osteoporosis was diagnosed when BMD was less than or equal to 2.5 standard deviations below that of a young, healthy adult reference population aged 20–30 years old.

### Slow gait

The general diagnostic criteria for sarcopenia is consistent with slow gait speed and objectively measured low lean mass. There was consensus that patient with measured gait speed less than 1 m/s was considered as slow gait[11]. A measured Walking Test Track was performed in NHANES to assess gait speed and the track is 20 feet long with a line at 8 feet. The operator calculated the time since the examinees touched the start line until they reached the finish line.

### Balance problem

The operator asked the respondents if they had dizziness, imbalance, and histories of falling in the past 12 months. Respondents who replied “yes” were considered having balance problem in our study.

### Low muscle strength

Muscle strength was estimated by detecting the isokinetic strength of the quadriceps muscle. A Kin Com MP isokinetic dynamometer (Chattanooga, TN) was used to assess the maximum voluntary concentric muscle force of the right knee extensor muscle with an angular velocity goal of 60 degrees/second[12].

### Covariates

The covariates included those taken from the analysis of the self-report demographic information including age, sex, smoking history and alcohol consumption. Medical history was also obtained from the participants included arthritis, congestive heart failure, coronary heart disease, angina pectoris, heart attack, stroke and moderate to vigorous recreational activity. Body mass index (BMI) was measured as the weight in kilograms divided by the square of height in meters. WC was estimated by asking the participant to stand stably and use a flexible, non-elastic anthropometric tape. Homeostasis model assessment for insulin resistance (HOMA-IR) was calculated by a formula: (fasting serum insulin [μU/ml] × fasting plasma glucose [mmol/l])/22.5[13]. The laboratory data analyzed in the study included levels of serum aspartate aminotransferase (AST), total cholesterol (TC), high density lipoprotein cholesterol (HDL-C), fasting plasma glucose (FPG), C-reactive protein (CRP), total calcium (Ca) and serum creatinine (Cr).

### Statistical analysis

All statistical data were analyzing by the Statistical Package for the Social Sciences, version18.0 (SPSS Inc., Chicago, IL, USA) for Windows. One-way analyses of variance and Pearson's chi-square tests were utilized to examine the differences between the groups in terms of demographic information, anthropometric parameters, and laboratory data. A two-sided *p*-value of ≤ 0.05 was regarded as the threshold for statistical significance. A linear regression model was performed for the association between number of MetS components and DMS, and the relationship between individual MetS component and DMS. We adjusted for pertinent variables in this linear regression model as follows: Model 1 = unadjusted; Model 2 = Model 1+ (age, gender, race-ethnicity). Model 3 = Model 2+ (BMI, AST, TC, FPG, HDL-C, Ca, and Cr). Model 4 = Model 3+ (arthritis, congestive heart failure, coronary heart disease, angina, heart attack, stroke, smoking, moderate to vigorous recreational activity). Pearson correlation was used to examine the correlation between HOMA-IR with components of DMS.

## Results

### Characteristics of participants with or without dysmobility syndrome

Table 1 summarized the characteristics of participants enrolled in the current study. The mean age was 68.94±6.89 in non-DMS and 73.39±7.77 years in DMS. We divided subjects into 2 groups with or without DMS according to the general criteria of DMS (Table 2). Age, CRP, FPG and medical history of arthritis were significantly greater in DMS participants than in non-DMS, however, BMI was significantly lower in DMS group.

**Table 1:** Characteristics of participants with or without dysmobility syndrome.

Variables	No-Dysmobility syndrome N = 1179	Dysmobility syndrome N = 581	P-value
Continuous Variables, mean (SD)			
Age (years)	68.94(6.89)	73.39(7.77)	<0.001
BMI (kg/m^2^)	28.28(4.82)	27.17(5.20)	<0.001
HOMA-IR	4.40(5.98)	4.79(7.22)	0.381
C-reactive protein (mg/dL)	0.43(0.63)	0.61(0.97)	<0.001
Glycohemoglobin (%)	5.85(1.05)	5.95(1.27)	0.083
AST (U/L)	24.42(8.24)	24.74(11.35)	0.498
Total cholesterol (mg/dL)	209.05(39.62)	209.44(38.45)	0.843
Serum glucose (mg/dL)	103.97(37.88)	108.03(42.71)	0.043
HDL (mg/dL)	52.40(16.29)	53.60(16.90)	0.153
Total Calcium (mg/dL)	9.44(0.40)	9.43(0.44)	0.855
Creatinine (mg/dL)	0.90(0.51)	0.91(0.58)	0.837
Categorical variables, n (%)			
Gender			<0.001
Male	644(54.6)	260(44.8)	
Female	535(45.4)	321(55.2)	
Race/ethnicity			<0.001
Mexican American	215(18.2)	131(22.5)	
Other Hispanic	38(3.2)	28(4.8)	
Non-Hispanic white	711(60.3)	344(59.2)	
Non-Hispanic black	193(16.4)	60(10.3)	
Other	22(1.9)	18(3.1)	
Medical condition			
Arthritis	468(39.7)	287(49.4)	<0.001
Congestive heart failure	44(3.7)	35(6.0)	0.468
Coronary heart disease	96(8.1)	54(9.4)	0.563
Angina	77(6.5)	47(8.1)	0.746
Heart attack	80(6.8)	47(8.1)	0.771
Stroke	6(0.5)	10(1.7)	0.765
Cigarette smoking	630(53.4)	314(54.0)	0.739
Moderate to vigorous recreational activity	540(45.8)	274(47.2)	0.591

BMI, body mass index; homeostatic model assessment for insulin resistance (HOMA-IR); AST, aspartate aminotransferase; HDL, high-density lipoprotein.

**Table 2:** Characteristics of participants with or without metabolic syndrome and dysmobility syndrome.

Variables	Non-Metabolic syndrome			Metabolic syndrome		
	Non-Dysmobility syndrome N = 3164	Dysmobility syndrome N = 889	P-value	Non-Dysmobility syndrome N = 3128	Dysmobility syndrome N = 1421	P-value
Continuous Variables, mean (SD)						
Age (years)	69.28(7.04)	73.17(7.96)	<0.001	68.33(6.60)	73.56(7.53)	<0.001
BMI (kg/m^2^)	26.83	25.38	<0.001	30.51(4.58)	29.30(4.81)	<0.001
HOMA-IR	2.61(1.97)	2.79(2.28)	0.346	6.83(8.34)	7.23(10.07)	0.680
C-reactive protein (mg/dL)	0.39(0.57)	0.66(1.19)	<0.001	0.49(0.73)	0.55(0.64)	0.325
Glycohemoglobin (%)	5.60(0.70)	5.61(0.82)	0.776	6.25(1.35)	6.37(1.60)	0.271
AST (U/L)	24.44(7.64)	25.27(12.68)	0.199	24.42(9.21)	23.92(9.51)	0.492
Total cholesterol (mg/dL)	206.43(35.92)	207.34(36.81)	0.713	212.67(43.12)	212.42(40.74)	0.938
Serum glucose (mg/dL)	94.93(21.32)	95.77(21.81)	0.566	118.27(51.69)	123.42(56.13)	0.219
HDL (mg/dL)	50.46(16.50)	60.27(16.75)	0.013	44.86(12.69)	45.75(13.65)	0.381
Total Calcium (mg/dL)	9.46(0.39)	9.44(0.47)	0.463	9.41(0.43)	9.45(0.38)	0.246
Creatinine (mg/dL)	0.91(0.48)	0.88(0.49)	0.401	0.88(0.36)	0.95(0.68)	0.045
Categorical variables, n (%)						
Gender			<0.001			0.208
Male	415(58.7)	144(46.8)		215(47.4)	107(42.3)	
Female	292(41.3)	164(53.2)		239(52.6)	146(57.7)	
Race/ethnicity			0.012			0.231
Mexican American	104(14.7)	64(20.8)		107(23.6)	171(24.2)	
Other Hispanic	19(2.7)	9(2.9)		18(4.0)	35(5.0)	
Non-Hispanic white	441(62.4)	190(61.7)		261(57.5)	405(57.3)	
Non-Hispanic black	128(18.1)	34(11.0)		61(13.4)	84(11.9)	
Other	15(2.1)	11(3.6)		7(1.5)	12(1.7)	
Medical condition						
Arthritis	282(39.9)	143(46.4)	0.052	179(39.4)	134(53.0)	0.002
Congestive heart failure	24(3.4)	21(6.8)	0.470	19(4.2)	13(5.1)	0.770
Coronary heart disease	51(7.2)	27(8.8)	0.141	44(9.7)	25(9.9)	0.741
Angina	41(5.8)	15(4.9)	0.141	35(7.7)	30(11.9)	0.933
Heart attack	44(6.2)	25(8.1)	0.596	33(7.3)	20(7.9)	0.302
Stroke	2(0.3)	4(1.3)	0.903	6(0.9)	4(2.0)	0.702
Cigarette smoking	379(53.6)	173(56.2)	0.641	243(53.5)	133(52.6)	0.896
Moderate to vigorous recreational activity	333(47.1)	139(45.1)	0.563	197(43.4)	123(48.6)	0.181

N, number; SD, standard deviation; BMI, body mass index; homeostatic model assessment for insulin resistance (HOMA-IR); AST, aspartate aminotransferase; HDL, high-density lipoprotein.

### Association between number of metabolic syndrome components and dysmobility syndrome

The association between number of MetS components and DMS was conducted by the multivariate linear regression model (Table 3). Participants with 3 components of MetS had association with the presence of DMS in Model 2, 3 and 4 and β coefficient were 0.102, 0.101 and 0.103 (95% confidence interval (CI): 0.012, 0.192, P<0.05; 0.006, 0.196, P<0.05; 0.008, 0.199, P<0.05, respectively). In all models, significant relationship were found between subjects with at least 4 components of MetS and the presence of DMS and β coefficient were 0.125, 0.145, 0.139 and 0.142 (95%CI: 0.034, 0.217, P<0.05; 0.050, 0.239, P<0.05; 0.032, 0.247, P<0.05; 0.035, 0.249, P<0.05, respectively). Notably, the increased number of MetS components had significant association with the occurrence of DMS.

**Table 3:** Regression coefficients of number of MetS components for dysmobility syndrome.

	Model 1		Model 2		Model 3		Model 4	
	β (95% CI)	P-value	β (95% CI)	P-value	β (95% CI)	P-value	β (95% CI)	P-value
Presence of MetS	0.053 (0.008, 0.998)	0.021	0.068 (0.021, 0.114)	0.004	0.058 (0.005, 0.111)	0.033	0.058 (0.005, 0.111)	0.034
Number of MetS components								
1	0.057(-0.031, 0.146)	0.205	0.044(-0.041, 0.129)	0.307	0.043(-0.042, 0.129)	0.321	0.047(-0.038, 0.132)	0.281
2	0.038(-0.048, 0.124)	0.389	0.066(-0.019, 0.152)	0.128	0.066(-0.021, 0.153)	0.139	0.068(-0.019, 0.156)	0.124
3	0.068(-0.020, 0.156)	0.130	0.102(0.012, 0.192)	0.027	0.101(0.006, 0.196)	0.038	0.103(0.008, 0.199)	0.033
≧4	0.125(0.034, 0.217)	0.008	0.145(0.050, 0.239)	0.003	0.139(0.032, 0.247)	0.011	0.142(0.035, 0.249)	0.009
P for trend	<0.001		<0.001		<0.001		<0.001	

Model 1 = unadjusted. Model 2 = Model 1+ age, race-ethnicity. Model 3 = Model 2+ (body mass index, aspartate aminotransferase, total cholesterol, serum calcium, creatinine). Model 4 = Model 3+ (arthritis, congestive heart failure, coronary heart disease, angina, heart attack, stroke, smoking, moderate to vigorous recreational activity)

### Association between individual metabolic syndrome component with dysmobility syndrome

In Table 4, we analyzed the relationship of individual MetS components and the presence of DMS by multi-modes adjustment and linear regression. We only observed that a strong positive association between elevated serum glucose levels and the occurrence of DMS achieved statistical significance in all models.

**Table 4:** Regression coefficients of individual component of metabolic syndrome for dysmobility syndrome.

	Model 1		Model 2		Model 3		Model 4	
	β (95% CI)	P-value	β (95% CI)	P-value	β (95% CI)	P-value	β (95% CI)	P-value
Components of metabolic syndrome								
High blood pressure	0.032(-0.013, 0.076)	0.162	-0.013(-0.056, 0.030)	0.565	-0.014(-0.058, 0.029)	0.509	-0.011(-0.054, 0.032)	0.623
Abdominal obesity	-0.012(-0.057, 0.034)	0.611	0.042(-0.016, 0.100)	0.157	0.037(-0.021, 0.096)	0.214	0.034(-0.024, 0.093)	0.251
Low HDL-C	0.027(-0.019, 0.074)	0.251	0.029(-0.017, 0.075)	0.212	0.026(-0.035, 0.087)	0.406	0.023(-0.038, 0.084)	0.465
High triglycerides	0.027(-0.018, 0.073)	0.236	0.043(-0.001, 0.087)	0.056	0.040(-0.009, 0.090)	0.112	0.040(-0.010, 0.090)	0.115
High glucose	0.084(0.039, 0.130)	<0.001	0.092(0.048, 0.137)	<0.001	0.082(0.029, 0.135)	0.003	0.083(0.030, 0.136)	0.002

Model 1 = unadjusted. Model 2 = Model 1+ age, race-ethnicity. Model 3 = Model 2+ (body mass index, aspartate aminotransferase, total cholesterol, serum calcium, creatinine). Model 4 = Model 3+ (arthritis, congestive heart failure, coronary heart disease, angina, heart attack, stroke, smoking, moderate to vigorous recreational activity)

### Correlation between HOMA-IR and components of dysmobility syndrome

In addition to low muscle mass (r = -0.121, P<0.05), there were no correlation between HOMA-IR and other components of DMS in subjects without MetS (Table 5). In the MetS population, HOMA-IR was correlated to body fat (r = 0.092, P<0.05), osteoporosis (r = -0.105, P<0.05) and balance (r = 0.105, P<0.05) analyzed by Pearson’s correlation coefficients.

**Table 5:** Associations between components of dysmobility syndrome and HOMA-IR and CRP.

Variables	Subgroup	Increased body fat		Low muscle mass		Slow gait		Low muscle strength		Osteoporosis		Balance problem	
		r	P-value	r	P-value	r	P-value	r	P-value	r	P-value	r	P-value
HOMA-IR	MetS	0.092	0.032	-0.085	0.050	0.079	0.075	-0.001	0.989	-0.105	0.014	0.105	0.013
	Non-MetS	0.058	0.125	-0.121	0.002	0.017	0.660	-0.021	0.634	-0.058	0.126	0.030	0.433
CRP	MetS	0.055	0.074	0.047	0.138	0.069	0.031	0.005	0.897	-0.048	0.124	0.010	0.743
	Non-MetS	0.039	0.144	0.056	0.036	0.078	0.004	0.067	0.032	0.046	0.085	0.039	0.137

CRP, C-reactive protein; homeostatic model assessment for insulin resistance (HOMA-IR); MetS, metabolic syndrome

## Discussion

In the present study, we observed the positive association between the increased number of MetS components and the presence of DMS among older adult population. Notably, patients with high serum glucose had significantly associated with the existence of DMS after multi-model adjustment. No significant difference was noted between other components of MetS and DMS. HOMA-IR, a method used to quantify insulin resistance and beta-cell function, was of paramount importance with individual components of DMS. To the best of our knowledge, this study was the first to examine the association between MetS and DMS which data was derived from the large-scale population survey.

Before the concept of DMS, several terms were applied to accurately identify older people most at risk of serious declines in health, independence, and function. Frailty, an age-related decline in multiple physiological domains, manifested an increased vulnerability to adverse health outcomes and resulted in high risk for falls, disability, hospitalization, and mortality[14]. MetS was consider as a risk factor for occurrence of the frailty in a sample of older noninstitutionalized individuals (Table 6) [15]. Rosenberg et al. described the origins of the term sarcopenia that revealed important changes in body composition and related functions[16]. In the last decade, some definitions for sarcopenia had been used to facilitate diagnosis both in clinical care and the ambulatory setting. European Working Group on Sarcopenia in Older People recommended using the presence of low muscle mass, low muscle function and physical disability[1]. In a cross sectional study of 8570 men aged 20–75 years, inverse association between muscle strength and MetS was observed [17]. Significant relationship of low muscle mass and MetS was reported in Asian population[18]. Increased rate of mobility dysfunction was suggested to be associated with MetS[19] and a linear increase in disability might be associated with the number of MetS components in an elderly population[20]. The direct impact of MetS on increased risk of sarcopenia was investigated in community-dwelling older adults[21]. Recently, osteosarcopenic obesity, a combined concept was published to identify the concurrent incident of impaired bone condition, reduced muscle mass and strength, and increased adiposity[2]. MetS was considered to be a risk factor for developing osteoporosis in men[22]. Insulin resistance was confirmed to lead to accelerated muscle loss and implied those with diabetes were likely to present with osteosarcopenic obesity[23]. Taking the above together, the domains of DMS were included the health problems and conditions of frailty, sarcopenia and osteosarcopenic obesity. It was consistent with our findings that the presence of MetS and increased number of MetS components had positive association with DMS.

**Table 6:** The associations among metabolic syndrome, frailty, sarcopenia, osteosarcopenia and dysmobility syndrome.

Date Authors	Aim of the study	Definition	Findings
2017 Our study	Association between metabolic syndrome (MetS) and DMS	Dysmobility present if ≧3 factors: osteoporosis, low lean mass, history of falls within the past year, slow gait speed, low grip strength, and high fat mass	A strong relationship between DMS and the presence of MetS and its components in elderly population, highlighting a possible mechanism through insulin resistance
2016 Viscogliosi	Association between MetS and frailty syndrome in older noninstitutionalized individuals	Frailty was defined based on following criteria were present: unintentional weight loss, self-reported exhaustion, weakness (grip strength), slow walking speed, and low physical activity.	MetS was associated with frailty. Such associations were not driven by specific altered components of MetS or by their sum.
2014 Ishii	Assessed the associations of MetS with sarcopenia in older adults	Diagnosis is based on documentation of criterion 1 plus (criterion 2 or criterion 3): low muscle mass, low muscle strength, low physical performance	MetS is positively associated with sarcopenia in older men, and central obesity is the main contributor to the association across sex and age.
2017 JafariNasabian	Osteosarcopenic obesity (OSO) syndrome and its individual components	The criteria for the diagnosis include the combination of its individual components: osteoporosis/osteopenia and/or osteopenic obesity, sarcopenia and/or sarcopenic obesity and increased adiposity.	OSO syndrome was cause by dysregulation of major metabolic pathways due to pro-inflammatory factors and endocrine imbalance such as low-grade chronic inflammation and insulin resistance.

Skeletal muscle was a major target of insulin and insulin receptors in the muscle played an important role in glucose regulation, and muscle is a major site of glucose disposal[24]. The development of DM and hyperglycemia had reported to be induced by obesity[25]. In a cross-sectional study, hyperglycemia was significantly associated with relatively lower lean muscle mass[26]. Hyperglycemia was an imperative characteristic of lower muscle strength examining from the Baltimore Longitudinal Study of Aging data[27]. The relationship between hyperglycemia and higher levels of inflammatory factors might be considered as a plausible explanation for alternation of body composition[28]. TNF-α, an important cytokine in skeletal muscle wasting and reduced muscle function[29], was involved with decline in grip strength and borderline associated with decline in knee extensor strength[28]. Diabetic neuropathy was a common complication in diabetes caused by hyperglycemia-induce oxidative stress in diabetic neurons and multiple biochemistry pathways[30]. It was also related to loss of foot muscle volume, muscle strength and motor dysfunction[31]. Patients with diabetes was associated with altered bone turnover and had increased risk for developing osteoporosis[32]. Chronic hyperglycemia was reported to result in bone loss by modulating osteoblast gene expression, function, and bone formation[33]. There were several underlying pathways for explaining the mechanism of bone formation loss. Wang et al. indicated that higher level of glucose could increase adipogenic and inhibit osteogenic differentiation by activating cAMP/PKA/ERK pathway in MG-63 cells then markedly suppressed cell growth, mineralization, and expression of various osteoblast-related markers[34]. A histomorphometric analysis revealed increases in osteoclast numbers and expression of osteoclastogenic mediators, including TNF-α, MCSF, RANKL and VEGF-A, which promoted osteoclastogenesis and diminished primary bone formation[35]. It was consistent with our study that hyperglycemia was significantly associated with DMS, consisted of six domains mentioned in above discussion.

In the present study, HOMA-IR was correlated to body fat, osteoporosis and balance problems in subjects with MetS and low muscle mass in participants without MetS. Insulin was a catabolic hormone, which induced the process of protein degeneration and synthesis in skeletal muscle[36]. Lee et al. demonstrated the relationship between greater lean muscle mass loss and insulin resistance as assessed by HOMA-IR in men without diabetes[26]. Inverse association was noted between insulin resistance and muscle mass resulted from alteration of insulin signaling which could lead to reduced muscle synthesis[37]. A potential mechanism for the effect of insulin resistance on skeletal muscle was that increased lipid accumulation in type 2 diabetes was related to reduced oxidative enzyme capacity[38], and defects in muscle lipid oxidation due to impaired mitochondria number or function could lead to this process. The association between insulin resistance and body fat had been reported in the past[39]. Mitochondria dysfunction was caused by insulin resistance and might contribute to free fatty acid and lipid accumulation[40]. Inflammation in adipose tissue, as suggested by the presence of macrophage in the form of crown-like structures, had been identified as a mediator of systemic insulin resistance[39]. Substantial evidence had supported that old people with DM had increased risk of fracture[41–43]. However, there were contradictory results for the association of insulin resistance and bone mineral density in patients with DM that a study reported increased bone mineral density[44] and another showed decreased[45]. In a previous study enrolled large scale Asian population, Shin et al. reported that HOMA-IR were inversely associated with BMD and osteoporosis and indicated insulin resistance was an adverse predictor for bone health[46]. Reduced bone mineral density had been represented in conditions related to insulin resistance, such as MetS[47] and increased fat mass[48]. It was compatible with our findings that HOMA-IR was correlated to osteoporosis in MetS group. Elevated levels of proinflammatory cytokines such as IL-6 as well as TNF-α were noted in subjects with DM and might induce bone loss by stimulating osteoclast activity[49]. Recent research demonstrated that the level of myokines was involved in the muscle contraction of regular physical activity, which was considered to be released from skeletal muscle[50]. Myokines regulated the lipid and glucose metabolism, and they were associated with insulin resistance through the control of inflammation. In older population, insulin resistance was considered as not only an early biological marker, but also a predictive and modifiable marker of mobility limitation[51]. It was reasonable that impaired physical function might arise from the joint of low muscle mass, reduced muscle strength, excess adiposity and adverse bone condition.

Although all efforts went into this study, there were several potential limitations should be noted. First, the present study was a cross sectional approach that no casual association between MetS and DMS. Future studies are warranted to assess the prospect by using longitudinal design with large sample size. Second, there was no muscle pathology from muscle biopsy reporting in our study, and we could not confirm the hypothesis. Third, sex difference was not analyzed during this study. Further studies should address the association of sex, MetS and DMS. Next, selection bias is existed in the present study. NHANES data are not obtained using a simple random sample. Rather, a complex, multistage, probability sampling design is used to select participants representative of the civilian, non-institutionalized US population. Oversampling of certain population subgroups is done to increase the reliability and precision of health status indicator estimates for these groups. In the present study, we included participants who had finished examinations of DMS: body composition measurement, bone mineral density measurement, gait speed test, and muscle strength measurement. There were 581 subjects with DMS and 1179 without DMS. A randomized controlled trial is needed for further research to examine the association between MetS and DMS. Finally, the generalization of the findings to other populations with different race/ethnicity may be limited.

## Conclusion

The present study highlighted a strong relationship between DMS and the presence of MetS and its components in elderly population. Notably, this dynamic interplay between DMS and MetS underlined the pathophysiology of insulin resistance. MetS aggravated inflammation and insulin resistance, which harbored a predisposing milieu for developing DMS. Our findings brought new insights for the development of this new field. Further researches should explore the underlying mechanisms for the clinical observations and therapeutic strategies to encounter the adverse health problems.

## Supporting information

S1 Data (RAR)
